# Targeted next-generation sequencing identifies clinically relevant somatic mutations in a large cohort of inflammatory breast cancer

**DOI:** 10.1186/s13058-018-1007-x

**Published:** 2018-08-07

**Authors:** Xu Liang, Sophie Vacher, Anais Boulai, Virginie Bernard, Sylvain Baulande, Mylene Bohec, Ivan Bièche, Florence Lerebours, Céline Callens

**Affiliations:** 1 Department of Breast Oncology, Key Laboratory of Carcinogenesis and Translational Research (Ministry of Education), Peking University Cancer Hospital & Institute, Beijing, China; 2 Pharmacogenomic Unit, Department of Genetics, Curie Institute, PSL Research University, 26 rue d’Ulm, 75005 Paris, France; 3 Clinic bioinformatic Unit, Department of Biopathology, Curie Institute, PSL Research University, Paris, France; 4 Institut Curie Genomics of Excellence (ICGex) Platform, Curie Institute, PSL Research University, Paris, France; 5 EA7331, Paris Descartes University, Sorbonne Paris Cité, Faculty of Pharmaceutical and Biological Sciences, Paris, France; 6 Department of Medical Oncology, Curie Institute, René Huguenin Hospital, Saint-Cloud, France

**Keywords:** Inflammatory breast cancer, Targeted NGS, Somatic mutation, Prognosis

## Abstract

**Background:**

Inflammatory breast cancer (IBC) is the most aggressive form of primary breast cancer. Using a custom-made breast cancer gene sequencing panel, we investigated somatic mutations in IBC to better understand the genomic differences compared with non-IBC and to consider new targeted therapy in IBC patients.

**Methods:**

Targeted next-generation sequencing (NGS) of 91 candidate breast cancer-associated genes was performed on 156 fresh-frozen breast tumor tissues from IBC patients. Mutational profiles from 197 primary breast tumors from The Cancer Genome Atlas (TCGA) were used as non-IBC controls for comparison analysis. The mutational landscape of IBC was correlated with clinicopathological data and outcomes.

**Results:**

After genotype calling and algorithmic annotations, we identified 392 deleterious variants in IBC and 320 variants in non-IBC cohorts, respectively. IBC tumors harbored more mutations than non-IBC (2.5 per sample vs. 1.6 per sample, *p* < 0.0001). Eighteen mutated genes were significantly different between the two cohorts, namely *TP53*, *CDH1*, *NOTCH2*, *MYH9*, *BRCA2*, *ERBB4*, *POLE*, *FGFR3*, *ROS1*, *NOTCH4*, *LAMA2*, *EGFR*, *BRCA1*, *TP53BP1*, *ESR1*, *THBS1*, *CASP8*, and *NOTCH1*. In IBC, the most frequently mutated genes were *TP53* (43.0%), *PIK3CA* (29.5%), *MYH9* (8.3%), *NOTCH2* (8.3%), *BRCA2* (7.7%), *ERBB4* (7.1%), *FGFR3* (6.4%), *POLE* (6.4%), *LAMA2* (5.8%), *ARID1A* (5.1%), *NOTCH4* (5.1%), and *ROS1* (5.1%). After grouping 91 genes on 10 signaling pathways, we found that the DNA repair pathway for the triple-negative breast cancer (TNBC) subgroup, the RTK/RAS/MAPK and cell cycle pathways for the HR^–^/HER2^+^ subgroup, the DNA repair, RTK/RAS/MAPK, and NOTCH pathways for the HR^+^/HER2^–^ subgroup, and the DNA repair, epigenome, and diverse pathways for the HR^+^/HER2^+^ subgroup were all significantly differently altered between IBC and non-IBC. *PIK3CA* mutation was independently associated with worse metastasis-free survival (MFS) in IBC since the median MFS for the *PIK3CA* mutant type was 26.0 months and for the *PIK3CA* wild type was 101.1 months (*p* = 0.002). This association was observed in TNBC (*p* = 0.04) and the HR^–^/HER2^+^ subgroups (*p* = 0.0003), but not in the HR^+^/HER2^–^ subgroup of IBC.

**Conclusions:**

Breast cancer-specific targeted NGS uncovered a high frequency of deleterious somatic mutations in IBC, some of which may be relevant for clinical management.

**Electronic supplementary material:**

The online version of this article (10.1186/s13058-018-1007-x) contains supplementary material, which is available to authorized users.

## Background

Inflammatory breast cancer (IBC) is a breast adenocarcinoma defined by a rapid onset of inflammatory signs involving at least one-third of the breast, such as erythema and edema (also known as ‘peau d’orange’) [1]. Although IBC is rare, constituting 1–5% of breast cancer cases, it harbors aggressive behavior with poor a prognosis and accounts for roughly 10% of breast cancer mortality annually [2]. Compared to non-IBC, IBC frequently presents resistance to conventional therapies and early recurrence. Although therapeutic progress in the past two decades in the context of non-IBC has also had a positive impact in women with IBC, with a more than 22-month improvement in median breast cancer-specific survival (BCSS) and a 14% improvement in 2-year BCSS [3], IBC is still a challenge for breast cancer physicians because of poor survival and lack of specific treatment. The clinical presentation and outcome of IBC are obviously different from those of non-IBC but there is no significant difference in treatment between IBC and stage III non-IBC. The poor understanding of the specific biological and molecular characteristics of IBC precludes specific therapeutic interventions. We urgently need to identify how and why IBC is distinct from non-IBC.

The ability to exploit the genetic information of a tumor for any clinical potential has only recently become evident. In this evidence-based precision medicine, genetic data have been exploited to identify therapies appropriate for an individual and has led to changes in drug oversight policy and the way certain drugs have been designated. As a special case of breast cancer mostly defined by clinical symptoms, IBC genome-specific maps are barely understood. Thus far, in previous studies, the IBC gene expression profiles demonstrated high transcriptional heterogeneity and heavy overall mutation burden compared with non-IBC [4, 5]. The largest molecular biology research on IBC mainly focused on the transcriptome and demonstrated the presence of molecular subtypes similar to those of non-IBC tumors, although with over-representation of human epidermal growth factor receptor 2 (HER2)-enriched tumors and a low prevalence of Luminal A tumors, and suggested the deregulation of the expression of few genes in IBC compared with non-IBC, in particular those involved in cell motility, invasion, inflammatory pathways, and transforming growth factor (TGF)Beta signaling [6–8]. Recently, some studies reported a higher frequency of *TP53*, *PIK3CA*, and *ERBB2* mutations in IBC than in non-IBC [9, 10], but these studies were performed in a small series and need further study to draw any conclusions.

Therefore, there is a need to extensively describe the genomic alterations in IBC to identify pathways involved in metastatic processes and drug resistance and to generate new treatment strategies for IBC patients. We have designed a breast cancer and targeted treatment-associated gene panel and performed targeted next-generation sequencing (NGS) in a large cohort of 156 IBC samples. Using the clinicopathological data and long-term survival follow-up, the association of the IBC mutational landscape with clinical outcomes was studied.

## Methods

### Samples

Tumor samples were collected from 156 women with IBC who underwent core biopsies at the Curie Institute/Rene Huguenin Hospital (Saint-Cloud, France) between 1988 and 2012. Each patient signed a written informed consent form and the study was approved by the Curie Institute/Rene Huguenin Hospital ethics committee. Tumor samples were immediately stored in liquid nitrogen after biopsy or surgery until DNA extraction. The samples analyzed contained more than 70% tumor cells.

Criteria for the diagnosis of IBC were the simultaneous presence of diffuse erythema and edema (peau d’orange) involving at least one-third of the breast with or without a measurable breast mass (staged T4d according to the AJCC classification) [1]. All patients with IBC tumors prospectively collected between 1988 and 2012 (with only 27 IBC samples collected between 1988 and 2003) received anthracycline-based ± taxane induction chemotherapy associated after 2003 with trastuzumab for HER2-positive tumors. Thirty-two of 43 HER2-positive patients (74.4%) received trastuzumab combined chemotherapy. Mastectomy with axillary node dissection was performed in all nonmetastatic patients following first-line systemic therapy. Radiation therapy was performed in all patients and hormone therapy was administered when indicated.

Public data for 197 invasive breast carcinomas from The Cancer Genome Atlas (TCGA) were used as a non-IBC dataset. This cohort was obtained using http://www.cbioportal.org [11, 12]. We extracted stage III and stage IV patients from 1105 samples in the clinical file downloaded from the TCGA data matrix (Breast invasive carcinoma, TCGA Provisional 2016), and we filtered out male patients, patients without completed cancer status information, and inflammatory breast cancer which was shown to be T4d. Of the 226 patients selected, somatic mutations detected by whole-exome sequencing were available for 197 patients and the clinical characteristics are shown in Additional file 1 (Table S1).

### Identification of breast cancer subtypes

Estrogen receptor (ER) and progesterone receptor (PR) status were determined by immunohistochemical (IHC) staining of paraffin-embedded tissue with monoclonal antibodies as part of the routine diagnostic procedure. Nuclear staining of 10% of the invasive cells was considered positive. Hormone receptor (HR) positivity was defined as ER and/or PR positivity. The HER2 status was determined by IHC staining and fluorescence in-situ hybridization (FISH). HER2 positivity was defined as 3+ IHC staining intensity (strong membranous staining in 10% of cells) or gene amplification (a HER2/CEP17 gene copy ratio of 2.0) using FISH. The subtypes were assessed for the primary tumor site.

### Somatic mutation data collection

Targeted NGS was applied to a custom-made panel of 91 ‘breast cancer-specific’ genes selected for their involvement in breast cancer. This BreastCurie panel was made up of the most frequently mutated genes (mutation frequency greater than 1%) in breast cancer from TCGA [13] and genes with potential therapeutic-targeted mutation based on the agreement of biologic specialists of the Institut Curie. The BreastCurie panel includes 91 genes (Additional file 2: Table S2) which were grouped into nine different signaling pathways: PIK3CA/AKT/mTOR, RTK/RAS/MAPK, cell cycle and apoptosis, DNA repair, NOTCH, ER, extracellular matrix, transcription, and epigenome. The genes *KEAP1*, *LDLRAP1*, *STMN2*, *MYO3A*, *VHL*, *AGTR2*, *CTNNB1*, *APC*, *SF3B1*, and *MYH9* all have different functionalities and were thus grouped into a pathway called “diverse”.

For each sample, coding exons and intron-exon boundaries of all genes were amplified using two ultra-high-multiplex polymerase chain reaction (PCR) primer pools (4834 amplicons) based on Ion AmpliSeq Targeted Sequencing Technology (ThermoFisher Scientific, USA). DNA libraries were prepared using the TruSeq nano DNA kit (Illumina, San Diego, CA, USA). Targeted NGS was performed on an Illumina Hiseq2500 sequencer according to the manufacturer’s instructions using the paired-end 120 nucleotide (PE120) sequencing mode. Sequence data were aligned to the human reference genome (hg19) using the Bowtie2 algorithm. The single nucleotide variants (SNVs) and indels were called using the GATK UnifiedGenotyper with default parameters. Known variants found in dbsnp129 and dbsnp137 with a variant allele frequency (VAF) superior to 1% (1000 g or ESP6500) were removed. Filtered retained variants had to have a total coverage depth of greater than or equal to 100 reads and a VAF of at least 5%. The sequencing results were analyzed for base substitutions, short insertions and deletions (INDELs), and copy number alterations (focal amplifications and homozygous deletions) as previously described [14, 15]. Further confirmation of detected variants was performed with a comparison to public databases (cbioportal, tumorportal), and potential pathogenicity was evaluated with four different public algorithms (Polyphen2, Sift, Mutation Assessor, Mutation Taster). We annotated all variants detected with the ‘treatment algorithms’ as previously described [14, 16], and which was performed in SHIVA [17], SAFIR01 [18], and SAFIR02 (trial in progress). Briefly, only hotspots missense, splice-site mutation revealing in-frame exon skipping, in-frame micro-deletions, or micro-insertions that were well established to be activating mutations should be considered functionally relevant for oncogenes. Meanwhile, for tumor suppressor gene, nonsense mutations, splice-site mutation, or frameshift insertion/deletions were considered pathogenic; missense mutations were considered relevant if they were established inactivating mutations in silico or in the literature [14]. Eventually, detected mutations were classified as pathogenic variants, unknown pathogenic variants, and nonpathogenic variants. The mutational profile of the 197 non-IBC cohort from TCGA was obtained after exclusion of all genes that were not included in our 91 genes panel. The same algorithms and classification were used to annotate the somatic variants of non-IBC cohort from TCGA.

### DNA copy number estimation

We estimated the copy number variations (CNV) using ONCOCNV, a computed method and software tool for high-quality base counting of the sequenced genes, as previously described [15]. Only mapping and base quality more than 20 were considered. The total number of reads covering each gene area was summarized and then normalized twice: first, by the total number of reads covering the analyzed sample, and then by the median coverage of the 155 other samples. We considered marked amplification if 80% of the captured gene areas had a normalized count of 2 or more. We considered homozygous deletion if 80% of the captured gene areas had a normalized count of 0.5 or less.

### Statistical analysis

Statistical analyses were performed using GraphPad Prism (version 5.01) software. The results were considered statistically significant at a *p* value < 0.05. We constructed contingency tables and performed a χ^2^ test for the association between clinical features and gene mutation or pathway alteration and to compare the mutation profiles between IBC and non-IBC patients, and Fisher’s exact tests were used when a cell contained less than five. Follow-up was measured from the date of diagnosis to the date of last news for patients without any event. Metastasis-free survival (MFS) was determined as the interval between initial diagnosis and detection of the first distant metastasis, with an interval less than 6 months being excluded. Survival distributions were estimated by the Kaplan-Meier method, and survival was compared between groups with the log-rank test. The *p* values were based on the Wald test, and patients with one or more missing data were excluded. The Cox proportional hazards regression model was used to assess prognostic significance in the multivariate analysis and the results are presented as hazard ratios. All statistical tests were two sided at the 5% level of significance.

## Results

### Patients characteristics

Pathological and clinical characteristics available for 156 IBC are provided in Table 1. All patients were female. Median age at IBC diagnosis was 53 years (age range 23–84 years). Median follow-up was 50.4 months (range 3.0 to 212.4 months) for IBC patients. Thirty-six of the 156 IBC patients (23.1%) had stage IV disease at diagnosis. Metastases were detected in 62.8% (98/156) of IBC patients and 26.9% (53/197) of non-IBC patients (*p* < 0.0001). Patients from the IBC and non-IBC cohorts were respectively classified into four subgroups according to hormone receptor (HR) and HER2 status. We also combined HR^+^/HER2^–^ and HR^+^/HER2^+^ to be HR^+^ (72 patients, 46.2%), triple-negative breast cancer (TNBC) and HR^–^/HER2^+^ to be HR^–^ (72 patients, 53.8%), and HR^–^/HER2^+^ and HR^+^/HER2^+^ to be HER2^+^ subtypes (43 patients, 27.6%) during the IBC analysis.

**Table 1:** Pathological and clinical characteristics of the inflammatory breast cancer (IBC) cohort

	IBC
Total, *n* (%)	156 (100)
Age (years)	
Median	53
Range	23–84
≤50, *n* (%)	63 (40.5)
>50, *n* (%)	93 (59.5)
Sex, *n* (%)	
Female	156 (100)
Male	0 (0)
Stage, *n* (%)	
III	120 (76.9)
IV	36 (23.1)
ER status, *n* (%)	
Negative	89 (57.1)
Positive	67 (42.9)
PR status, *n* (%)	
Negative	111 (71.2)
positive	45 (28.8)
Her2 status, *n* (%)	
Negative	113 (72.4)
Positive	43 (27.6)
Subgroups, *n* (%)	
TNBC	51 (32.7)
HR^–^/Her2^+^	33 (21.2)
HR^+^/Her2^–^	62 (39.7)
HR^+^/Her2^+^	10 (6.4)
Distant metastases, *n* (%)	
Yes	98 (62.8)
No	58 (37.2)
SBR histological grade, *n* (%)	
I	2 (1.3)
II	63 (40.4)
III	91 (58.3)

*ER* estrogen receptor, *Her2* human epidermal growth factor receptor 2, *HR* hormone receptor, *PR* progesterone receptor, *SBR* Scarf Bloom Richardson classification, *TNBC* triple-negative breast cancer

### IBC mutation profiling

**Fig. 1:**
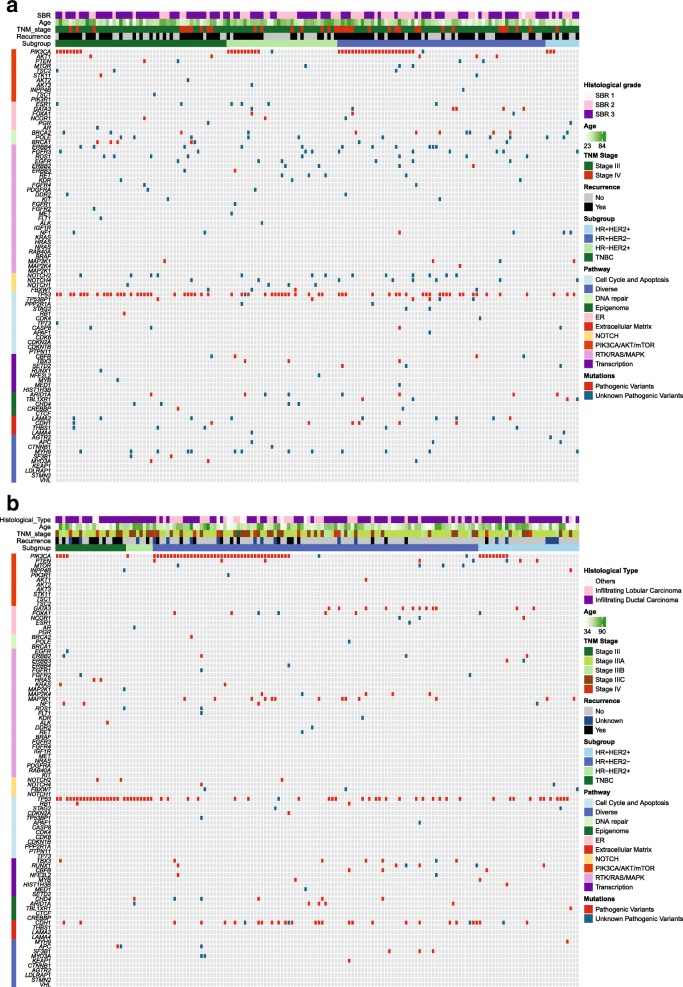
Deleterious somatic mutations of IBC tumors and non-IBC tumors. **a** Deleterious somatic mutations of 156 IBC tumors. **b** Deleterious somatic mutations of 197 non-IBC tumors. Tumors with available mutation data are grouped by four molecular subtypes along the *x* axis, with the *x* axis also showing clinic characteristics for each tumor; the 91 genes of BreastCurie panel are enriched to 10 signaling on the *y* axis. The somatic mutations of each tumor are indicated by colored boxes: red boxes indicate pathogenic variants, and green boxes indicate unknown pathogenic variants. ER estrogen receptor, HER2 human epidermal growth factor receptor 2, HR hormone receptor, SBR Scarff Bloom Richardson classification, TNBC triple-negative breast cancer, TNM tumor node metastasis

Among the 91 sequenced genes from 156 IBC samples, we detected 777 somatic mutations comprising 733 point mutations and 44 indels. Point mutations included 666 missense, 59 nonsense, and 8 splice-site mutations, and the 44 indels included 28 frame-shift and 16 in-frame. They corresponded to 516 different mutations in 84 genes, and 152 samples exhibited at least one mutation. After annotation of all these mutations, 392 mutations in 73 genes on 144 samples were predicted to have high probability of being deleterious, and were thus classified as pathogenic variants and unknown pathogenic variants (Fig. 1a). In the non-IBC, a total of 320 deleterious mutations in 56 genes on 146 samples was identified (Fig. 1b). All pathogenic variants and unknown pathogenic variants were considered as mutations for further research.

### Comparison of mutation frequency between IBC and non-IBC

**Fig. 2:**
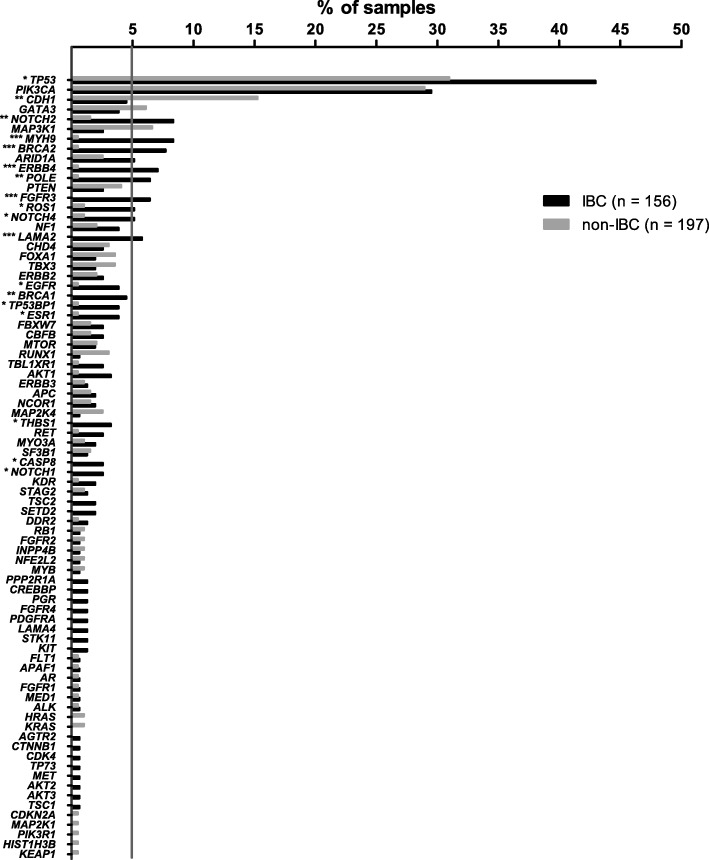
Comparison of somatic mutation frequency between inflammatory breast cancer (IBC) and non-IBC. Data show the percentage of samples with somatic mutations on our 91 gene panel; the gray bars indicate non-IBC, the black bars indicate IBC; *p < 0.05, **p < 0.01, ***p < 0.001

The average number of mutations per sample was higher in IBC than in non-IBC (2.5 vs. 1.6, *p* = 0.0009). We observed a significantly different mutation frequency between IBC and non-IBC patients for the following genes: *TP53*, *CDH1*, *NOTCH2*, *MYH9*, *BRCA2*, *ERBB4*, *POLE*, *FGFR3*, *ROS1*, *NOTCH4*, *LAMA2*, *EGFR*, *BRCA1*, *TP53BP1*, *ESR1*, *THBS1*, *CASP8*, and *NOTCH1*. All genes, except *CDH1* which was less frequently mutated in IBC, were more frequently mutated in IBC compared with non-IBC (Fig. 2). The most frequently mutated genes in IBC were *TP53* (43.0%), *PIK3CA* (29.5%), *MYH9* (8.3%), *NOTCH2* (8.3%), *BRCA2* (7.7%), *ERBB4* (7.1%), *FGFR3* (6.4%), *POLE* (6.4%), *LAMA2* (5.8%), *ARID1A* (5.1%), *NOTCH4* (5.1%), and *ROS1* (5.1%) (Fig. 2). A comparison was made of the mutated genes between IBC and non-IBC according to the four subgroups. Gene mutation frequencies were not significantly different in the triple-negative IBC and non-IBC subgroups (Additional file 3: Figure S1a). In the HR^–^/HER2^+^ subgroup, less mutations on *TP53* were detected in IBC (Additional file 3: Figure S1b). In the HR^+^/HER2^–^ subgroup (Additional file 3: Figure S1c), genes such as *BRCA2*, *NOTCH2*, *ERBB4*, *FGFR3*, and *LAMA2* (all *p* value less than 0.01), and *TP53*, *NOTCH4*, *TP53BP1*, *MYH9*, and *EGFR* (all *p* value less than 0.05) were more frequently mutated in IBC. In the HR^+^/HER2^+^ subgroup (Additional file 3: Figure S1d), *POLE* was found to be more significantly highly mutated in IBC than in non-IBC.

**Fig. 3:**
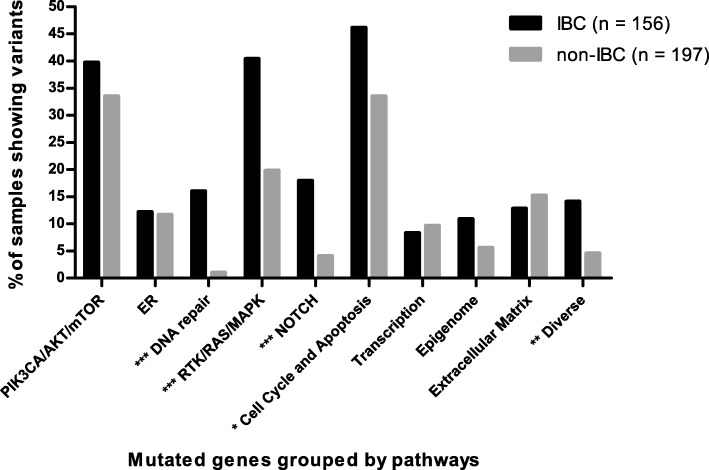
Comparison of somatic mutation frequency in inflammatory breast cancer (IBC) and non-IBC grouped by biological pathways. Data show the percentage of samples with alterations on 10 biological pathways; the gray bars indicate non-IBC, the black bars indicate IBC; *p < 0.05, **p < 0.01, ***p < 0.001

For a better understanding of IBC tumorigenesis, the 91 genes were categorized in 10 different signaling pathways and the pathway was considered altered when at least one gene of the pathway was mutated (Fig. 3). Alteration in DNA repair, RTK/RAS/MAPK, NOTCH, cell cycle and apoptosis, and diverse pathways showed significant differences between the two cohorts (*p* < 0.0001, *p* < 0.0001, *p* < 0.0001, *p* = 0.02, and *p* = 0.002, respectively). Furthermore, we compared alterations of these pathways according to subgroups (Additional file 4: Figure S2). In TNBC, the signaling pathway of DNA repair was more frequently altered in IBC than in non-IBC (*p* = 0.02) (Additional file 4: Figure S2a). In the HR^–^/HER2^+^ subgroup, the RTK/RAS/MAPK pathway was significantly more altered in IBC (*p* = 0.02), but the cell cycle and apoptosis pathway was more altered in non-IBC (*p* = 0.03) (Additional file 4: Figure S2b). IBC harbored more mutations in the DNA repair, RTK/RAS/MAPK, NOTCH, and cell cycle pathways in the HR^+^/HER2^–^ subgroup than in non-IBC (*p* = 0.0001, *p* = 0.003, *p* = 0.001, and *p* = 0.04, respectively) (Additional file 4: Figure S2c), and DNA repair, epigenome and diverse pathways were also found to be more altered in the HR^+^/HER2^+^ subgroup of IBC (*p* = 0.007, *p* = 0.02, and *p* = 0.02, respectively) (Additional file 4: Figure S2d).

Several pathways such as PIK3CA/AKT/mTOR, DNA repair, RTK/RAS/MAPK, NOTCH, and ER include targeted genes. The well-known targeted genes of RTKs and NOTCH families were frequently mutated in IBC. Intriguingly, more unknown pathogenic variants of *ERBB4*, *FGFR3*, *ERBB3*, and *PDGFRA* were detected on the catalytic domain of tyrosine kinase, and we also detected unknown pathogenic variants of *NOTCH4* on the ankyrin repeat domain in several IBC patients (Fig. 1a). Our results thus demonstrated a genomic instability of the IBC cell surface, and we are continuing to explore whether those frequent unknown pathogenic mutation of RTKs on *ERBB4*, *FGFR3*, *EGFR*, and *ERBB2* are functional and provide potential therapeutic targets in further research.

### DNA copy number alterations in IBC

We applied ONCOCNV to calculate the DNA copy number alterations (CNAs) for 156 IBC samples (Additional file 5: Figure S3). In 44 IBC samples with HER2-positive status detected via IHC or FISH, 38 samples were called as having *ERBB2* amplification with ONCOCNV calculation (86.4% concordance was achieved). The rate of *ERBB2* and *MED1* coamplification was 57.1% in IBC, which is similar to that reported in a non-IBC study [19]. The other frequently amplified genes were observed in *FGFR1* in 10.8%, *EGFR* in 4.4%, and *DDR2* in 3.2% of IBC samples. The frequent deletions were found in *RB1* in 5.1%, *STAG2* in 3.8%, and *CDKN2A* and *MAP3K1* in 3.2% of IBC.

### Survival analysis of IBC

**Fig. 4:**
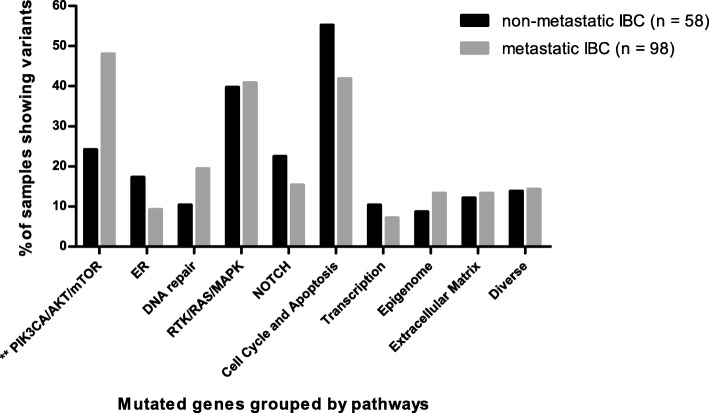
Comparison of biological pathway between non-metastatic inflammatory breast cancer (IBC) and metastatic IBC. Data show the percentage of samples with alterations on 10 biological pathways; the gray bars indicate metastatic IBC, the black bars indicate non-metastatic IBC; *p < 0.05, **p < 0.01, ***p < 0.001

We compared the gene mutation frequency in 10 pathways between 98 metastatic IBC and 58 non metastatic IBC (Fig. 4). PIK3CA/AKT/mTOR was the only pathway showing a significant difference, which was more frequently altered in metastatic IBC compared with nonmetastatic IBC (48.0% vs. 24.1%, *p* = 0.003). Regarding subgroups, a significant difference was found for this pathway in HR^+^/HER2^–^ (58.1% vs. 26.3%, *p* = 0.02). A higher mutation trend is shown in the three other subgroups. Among the 11 genes of the PIK3CA/AKT/mTOR pathway, *PIK3CA* mutations were highly enriched in the metastatic IBC group (38.8% vs. 13.8%, *p* = 0.0009).

**Fig. 5:**
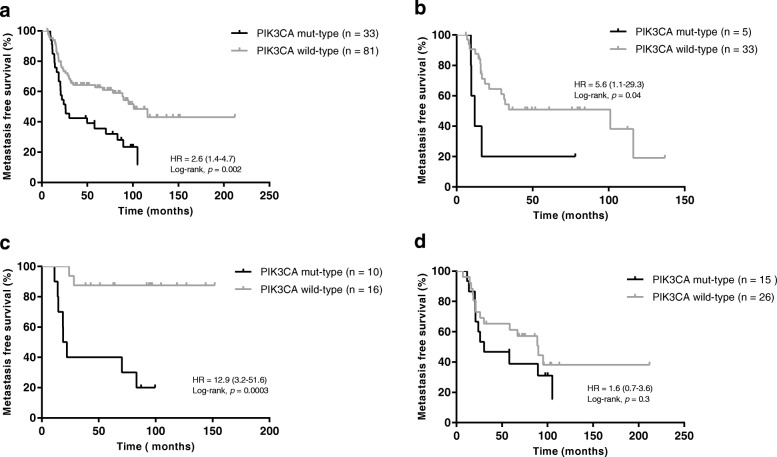
MFS curves of IBC patients stratified by *PIK3CA* mutation. **a** Kaplan-Meier estimates of MFS according to *PIK3CA* mutations in total IBC patients (*n* = 114); **b** Kaplan-Meier estimates of MFS according to *PIK3CA* mutations in the TNBC subgroup (*n* = 38); **c** Kaplan-Meier estimates of MFS according to *PIK3CA* mutations in the HR^–^/HER2^+^ subgroup (*n* = 26); **d** Kaplan-Meier estimates of MFS according to *PIK3CA* mutations in the HR^+^/HER2^–^ subgroup (*n* = 41). HR hazard ratio

Among 120 stage III IBC patients, MFS was available for 115 patients, and one patient was excluded because of disease progression within 6 months. We assessed the relation of *PIK3CA* mutation status with MFS in 114 patients. The median survival was 83.1 months (range 6.2 to 212.1 months). Univariate and multivariate analyses are reported in Table 2. The *PIK3CA* genotype was the only marker significantly associated with MFS in IBC patients (hazard ratio = 2.6, 95% confidence interval (CI) 1.4–4.7, *p* = 0.002; Fig. 5a). The median MFS for the *PIK3CA* mutant-type was 26.0 months and for the *PIK3CA* wild-type it was 101.1 months. Regarding the subgroups, *PIK3CA* mutation was associated with MFS in triple-negative (Fig. 5b) and HR^–^/HER2^+^ (Fig. 5c) subgroups, with hazard ratios for *PIK3CA* mutant-type of 5.6 and 12.9, respectively. A high frequency of *PIK3CA* mutation was observed in the HR^+^/HER2^–^ IBC subgroup; however, no association between *PIK3CA* mutant-type and MFS was found (hazard ratio = 1.5, 95% CI 0.7–3.6, *p* = 0.3; Fig. 5d). The number of cases was too small to interpret the results for the HR^+^/HER2^+^ subgroup. We combined subgroups for further analysis of prognostic impact of *PIK3CA* mutation, and the *PIK3CA* mutant-type was found to significantly associated with worse MFS in HR^–^ and HER2^+^ subgroups; no association was found in the HR^+^ subgroup (Additional file 6: Figure S4).

**Table 2:** Correlation of *PIK3CA* mutation and classic clinical characteristics with metastasis-free survival (MFS) in primary inflammatory breast cancer (IBC) patients with stage III disease (*n* = 114)

Parameters	*n*	Univariate analysis		Multivariate analysis	
		HR (95% CI)	*p* ^a^	HR (95% CI)	*p* ^b^
Age		0.8 (0.5–1.1)	0.6	1.0 (0.5–1.7)	0.9
≤50 years	48				
>50 years	66				
ER status		0.9 (0.6–1.6)	0.8	1.0 (0.5–2.0)	1.0
Negative	67				
Positive	47				
PR status		1.3 (0.8–2.3)	0.3	1.7 (0.8–3.5)	0.1
Negative	82				
Positive	32				
Her2 status		1.7 (1.0–2.8)	0.1	2.0 (1.1–3.8)	0.03
Negative	79				
Positive	35				
SBR grade		1.2 (0.7–2.0)	0.2	1.2 (0.7–2.2)	0.5
III	71				
I and II	43				
*PIK3CA*		2.6 (1.4–4.7)	0.002	2.7 (1.5–4.7)	0.001
Mut-type	33				
Wild-type	81				

*CI* confidence interval, *ER* estrogen receptor, *Her2* human epidermal growth factor receptor 2, *HR* hazard ratio, *PR* progesterone receptor, *SBR* Scarf Bloom Richardson classification

^a^ Log-rank test

^b^ Cox multivariate analyses

## Discussion

A key purpose of precision cancer medicine is to tailor clinical management based on the specific events that are relevant to tumor development and progression. The high frequency of clinically relevant genomic alterations in IBC when sequenced with a targeted NGS raises the possibility that targeted therapies may be developed for patients with this highly aggressive form of breast cancer. First, a heavy mutation burden was found in IBC tumors. This could be the hallmark of increased genomic instability correlating with tumor aggressiveness. *TP53* was the most frequently mutated gene, in accordance with previous studies on IBC [7, 10, 20]. There were 12 genes with more than 5% mutation frequencies in IBC: *TP53*, *PIK3CA*, *MYH9*, *NOTCH2*, *BRCA2*, *ERBB4*, *FGFR3*, *POLE*, *LAMA2*, *ARID1A*, *NOTCH4*, and *ROS1*. For *TP53*, *PIK3CA*, and *BRCA2*, high mutation rates in IBC have been also reported by other groups. In contrast, we did not detect higher frequent mutation of *ERBB2*, *RB1*, or *NOTCH1* [7, 9, 10].

Comparative analysis of biology pathways between IBC and non-IBC revealed high mutation frequencies of genes in DNA repair, NOTCH, and RTK/RAS/MAPK pathways that could be clinically relevant. The alteration of *BRCA1/BRCA2/POLE* genes of the DNA repair pathway was independent of molecular subtypes, so PARP inhibitor may be especially evaluated in IBC [21]. To the best of our knowledge, this is the first time that *POLE* has been detected frequently mutated in IBC. The correlation of *POLE* mutation with PD1/PD-L1 immunotherapy in colorectal cancer and endometrial cancer [22–24] leads us towards further research to explore whether there is a treatment option for immunotherapy in *POLE*-mutated IBC. Note that POLE-detected variants are not hotspots pathogenic variants that have already been described. *NOTCH1/2/4* and *FBWX7* genes were more frequently mutated in each subgroup of IBC compared with non-IBC. A preclinical study in IBC showed that a gamma secretase inhibitor, RO4929097, was able to block the Notch signaling and to attenuate the stem-like phenotype of IBC cells and regulate the inflammatory environment [25]. Targeting the Notch pathway might be an option for IBC treatment. Receptor tyrosine kinases (RTKs) are frequently activated in cancer cells and therefore have become the target of numerous treatments. The BreastCurie gene panel included most targetable RTK genes and we found that IBC carried higher frequencies of unknown pathogenic variants of RTKs than non-IBC. Higher gene instability due to DNA repair dysfunction may promote variants of unknown significance in IBC but we cannot exclude that other unknown mechanisms are also implicated. Activation of downstream pathways of RTKs, such as ERBB2, EGFR, and IGF1R, has been proven to be related to tumor cell anoikis resistance, and IBC cells have been associated with more evasion of anoikis [26, 27] which is consistent with our findings. We now aim to explore whether the frequent unknown pathogenic mutations of RTKs on *ERBB4*, *FGFR3*, *EGFR*, and *ERBB2* reported in the present study are potential therapeutic targets.

Compared with the breast cancer literature [10, 12, 13, 28], we did not find IBC-specific amplified genes with our gene panel. Unfortunately, some frequent CNAs of breast cancer (e.g., *MYC*, *CCND1* amplification) reported previously were not included in our gene panel. We detected *STAG2* deletion, a tumor suppressor gene coding cohesion protein, in 3.7% of IBC. *STAG2* loss of function was reported in different cancers but not in IBC [29]. However, ONCOCNV did not compute allele frequencies, which may affect the precision of the method in admixed data [15].

Our study demonstrates that *PIK3CA* gene mutations and PIK3CA/AKT/mTOR pathway alteration were very common events in IBC. *PIK3CA* gene mutations were especially observed in luminal and HER2-positive subtypes, and mainly located in hotspots of the helical domain and the catalytic domain, similar to non-IBC in previous reports [13, 30]. Recently, a large pooled analysis of more than 10,000 early-stage breast cancer patients reported that PIK3CA-mutated tumors are associated with a better prognosis [31]. However, this good prognostic effect was observed in HR^+^/HER2^–^ and TNBC subtypes, but not in the HER2^+^ subtype where *PIK3CA* mutations were associated with a worse overall survival. Interestingly, in our IBC cohort, *PIK3CA* mutation was a poor prognostic factor for MFS for HER2^+^ and TNBC subtypes, whereas no prognostic value was found in the HR^+^/HER2^–^ subtype. Of note, the prognostic effect was weak in the TNBC subtype of IBC since *PIK3CA* mutations were rare in this subtype and our TNBC cohort was small. For the HER2^+^ subtype, previous studies reported that *PIK3CA* mutations were associated with adverse prognosis in non-IBC, but results were not conclusive [31–34]. As *PIK3CA* mutation could lead to resistance to anti-HER2 treatments [34, 35], we checked that the percentage of patients in our IBC cohort receiving trastuzumab combined with chemotherapy was balanced in both *PIK3CA* genotypes (71.4% in mutant type, 75.9% in wild type). Therefore, the association between *PIK3CA* mutation and worse MFS in IBC may be reliable. For the HR^+^/HER2^–^ subtype, the prognostic difference regarding *PIK3CA* genotype between IBC and non-IBC may reflect the influence of the PI3K pathway in the two distinct biological environments of IBC and non-IBC, and we presume interactions between ER and PI3K pathways are different. We know that PI3K inhibitors have been investigated in many breast cancer trials and have shown promising results in ER-positive endocrine therapy-refractory breast cancer [36], but no clinical trials have been performed specifically in IBC to date. The association of *PIK3CA* mutations with worse MFS in IBC should draw our attention to the role of the PI3K pathway in this aggressive and treatment-refractory form of breast cancer. Further experimental research to explore the PI3K pathway in IBC is therefore required.

## Conclusions

Overall, IBC is the most aggressive form of breast cancer frequently refractory to conventional therapy and suffers from the lack of a specific treatment. Our study using targeted NGS analysis revealed a high frequency of somatic mutations, in particular in DNA repair, Notch signaling, and RTKs genes, that may guide switching from conventional therapy to targeted agents in IBC. In contrast to non-IBC patients, *PIK3CA* mutation was associated with a poor outcome in IBC patients. These findings encourage clinical trials with targeted therapies that may provide clinical benefit to IBC patients.

## Additional files

Additional file 1: **Table S1.** Pathological and clinical characteristics of non-IBC cohorts. (PDF 169 kb)

Additional file 2: **Table S2.** BreastCurie gene panel for targeted NGS. (PDF 411 kb)

Additional file 3: **Figure S1.** Comparison of somatic mutation frequency between IBC and non-IBC in four subgroups. (a) The percentage of samples with somatic mutation in the TNBC subgroup; (b) the percentage of samples with somatic mutation in the HR^–^/HER2^+^ subgroup; (c) the percentage of samples with somatic mutation in the HR^+^/HER2^–^ subgroup; (d) the percentage of samples with somatic mutation in the HR^+^/HER2^+^ subgroup. The gray bars indicate non-IBC, the black bars indicate IBC; *p < 0.05, **p < 0.01, ***p < 0.001. (PDF 52 kb)

Additional file 4: **Figure S2.** Comparison of biological pathway between IBC and non-IBC in four subgroups. (a) The percentage of samples with alteration on 10 biological pathways in the TNBC subgroup; (b) the percentage of samples with alteration on 10 biological pathways in the HR^–^/HER2^+^ subgroup; (c) the percentage of samples with alteration on 10 biological pathways in the HR^+^/HER2^–^ subgroup; (d) the percentage of samples with alteration on 10 biological pathways in the HR^+^/HER2^+^ subgroup. The gray bars indicate non-IBC, the black bars indicate IBC; *p < 0.05, **p < 0.01, ***p < 0.001. (PDF 41 kb)

Additional file 5: **Figure S3.** DNA copy number alterations in the IBC cohort. The genes with DNA copy number alterations are grouped along the *x* axis, the percentage of samples with DNA copy number alterations shown on the *y* axis, DNA amplifications are indicated by black bars above the *x* axis, and DNA deletions are indicated by gray bars below the *x* axis. (PDF 164 kb)

Additional file 6: **Figure S4.** MFS curves stratified by *PIK3CA* mutation in three subgroups of IBC patients. (a) Kaplan-Meier estimates of MFS according to *PIK3CA* mutations in patients of the HR^–^ subgroup, (b) Kaplan-Meier estimates of MFS according to *PIK3CA* mutations in patients of the HER2^+^ subgroup, (c) Kaplan-Meier estimates of MFS according to *PIK3CA* mutations in patients of the HR^+^ subgroup. (PDF 42 kb)

## Abbreviations

BCSS: Breast cancer-specific survival
BRCA1/2: Breast cancer 1/2
ER: Estrogen receptor
FISH: Fluorescence in-situ hybridization
HER2: Human epidermal growth factor receptor 2
HR: Hormone receptor
IHC: Immunohistochemical
MFS: Metastasis-free survival
mTOR: Mammalian target of rapamycin
NGS: Next-generation sequencing
NOTCH1/2/4: Neurogenic locus notch homolog protein 1/2/4
PI3K: Phosphatidylinositol 3-kinase
PIK3CA: Phosphatidylinositol 3-kinase: catalytic: alpha polypeptide gene
POLE: Polymerase (DNA) epsilon
PR: Progesterone receptor
RTK: Receptor tyrosine kinase
SBR: Scarff Bloom Richardson classification
TCGA: The Cancer Genome Atlas
TNBC: Triple-negative breast cancer
