# Supplementary material for: Targeted next-generation sequencing identifies clinically relevant somatic mutations in a large cohort of inflammatory breast cancer
Source: Breast Cancer Res. 2018 Aug 7;20:88. doi: 10.1186/s13058-018-1007-x (PMC6081877; doi:10.1186/s13058-018-1007-x)
Supplement: Supplementary file 1 — Table S1. Pathological and clinical characteristics of non-IBC cohorts. (PDF 169 kb) [file 13058_2018_1007_MOESM1_ESM.pdf]

Table S1. Pathological and clinical characteristics of non-IBC cohorts

	Non-IBC (%)
Total	197 (100)
Age(years)	
Median	59
Range	34-90
≤ 50	62 (31.5)
> 50	135 (68.5)
Sex	
Female	197 (100)
Male	0 (0)
Stage	
IIIA	114 (57.9)
IIIB	19 (9.6)
IIIC	52 (26.4)
IV	12 (6.1)
ER status	
Negative	39 (19.8)
Positive	158 (80.2)
PR status	
Negative	61 (31.0)
positive	136 (69.0)
Her2 status	
Negative	150 (76.1)
Positive	47 (23.9)
Subgroups	
TNBC	28 (14.2)
HR-/Her2+	9 (4.6)
HR+/Her2-	122 (61.9)
HR+/Her2+	38 (19.3)
Distant metastases	
Yes	53 (26.9)
No	144 (73.1)
Histologic type	
IDC	133 (67.5)
ILC	50 (25.4)
Others	14 (7.1)

IDC: invasive ductal carcinoma; ILC: invasive lobular carcinoma
