# Supplementary material for: Targeted next-generation sequencing identifies clinically relevant somatic mutations in a large cohort of inflammatory breast cancer
Source: Breast Cancer Res. 2018 Aug 7;20:88. doi: 10.1186/s13058-018-1007-x (PMC6081877; doi:10.1186/s13058-018-1007-x)
Supplement: Supplementary file 2 — Table S2. BreastCurie gene panel for targeted NGS. (PDF 411 kb) [file 13058_2018_1007_MOESM2_ESM.pdf]

Table S2. BreastCurie gene panel for targeted NGS

Gene name	NM_	Pathway
CDKN1B	004064.4	Cell cycle and apoptosis
PPP2R1A	014225.5	
PTPN11	002834.3	
RB1	000321.2	
TP53	000546.4	
STAG2	006603.4	
TP53BP1	005657.2	
CDK4	000075.3	
CDK6	001259.6	
TP73	005427.3	
CDKN2A	000077.4	
APAF1	181861.1	
CASP8	001228.4	
FBXW7	033632.3	NOTCH
NOTCH1	017617.3	
NOTCH2	024408.3	
NOTCH4	004557.3	
ARID1A	006015.4	Epigenome
CTCF	006565.3	
TBL1XR1	024665.4	
CREBBP	004380.2	
CHD4	001273.2	
ESR1	000125.3	ER
FOXA1	004496.3	
GATA3	002051,2	
NCOR1	006311.3	
PGR	000926.4	
AR	000044.3	
BRAF	004333.4	RTK/RAS/MAPK
EGFR	005228.3	
FGFR1	023110.2	
FGFR2	000141.4	
FGFR3	000142.4	
FGFR4	002011.4	
ERBB2	004448,3	
ERBB3	001982.3	
ERBB4	005235.2	
HRAS	005343.2	
IGF1R	000875.4	
KRAS	004985.4	
MAP2K1	002755.3	
MAP2K4	003010.3	

Gene name	NM_	Pathway
MAP3K1	005921.1	RTK/RAS/MAPK
MET	000245.2	
NF1	000267.3	
NRAS	002524.4	
PDGFRA	006206.4	
RAB40A	080879.2	
RET	020975.4	
ROS1	002944.2	
FLT1	002019.4	
KDR	002253.2	
ALK	004304.4	
DDR2	006182.2	
KIT	000222.2	
THBS1	003246.2	Extracellular matrix
CDH1	004360.3	
LAMA2	000426.3	
LAMA4	002290.4	
AKT1	005163.2	PIK3CA/AKT/mTOR
AKT2	001626.5	
AKT3	005465.4	
INPP4B	003866.2	
MTOR	004958.3	
PIK3CA	006218.2	
PIK3R1	181523.2	
PTEN	000314.4	
STK11	000455.4	
TSC1	000368.4	
TSC2	000548.3	
BRCA1	007294.3	DNA repair
BRCA2	000059.3	
POLE	006231.2	
RUNX1	001754.4	Transcription
TBX3	016569.3	
NFE2L2	006164.3	
MYB	005375.2	
HIST1H3B	003537.3	
SETD2	014159.6	
MED1	004774.3	
CBFB	001755.2	
KEAP1	012289.3	Diverse
LDLRAP1	015627.2	
STMN2	007029.3	
MYO3A	017433.4	

Gene name	NM_	Pathway
VHL	000551.3	Diverse
AGTR2	000686.4	
CTNNB1	001904.3	
APC	000038.5	
SF3B1	012433.2	
MYH9	002473.4	
