# Supplementary figures and images for: Targeted next-generation sequencing identifies clinically relevant somatic mutations in a large cohort of inflammatory breast cancer
Source: Breast Cancer Res. 2018 Aug 7;20:88. doi: 10.1186/s13058-018-1007-x (PMC6081877; doi:10.1186/s13058-018-1007-x)

S1a

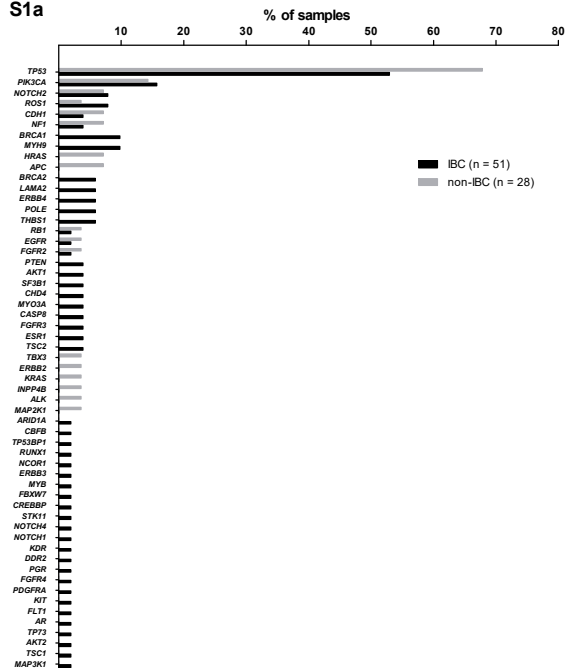

S1b

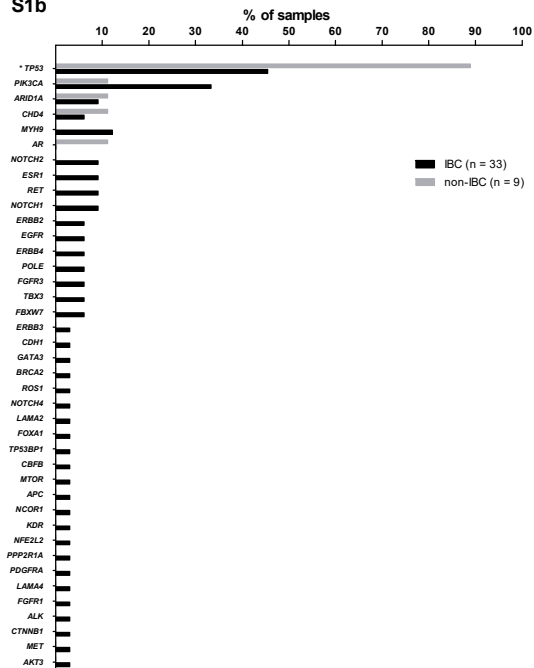

S1c

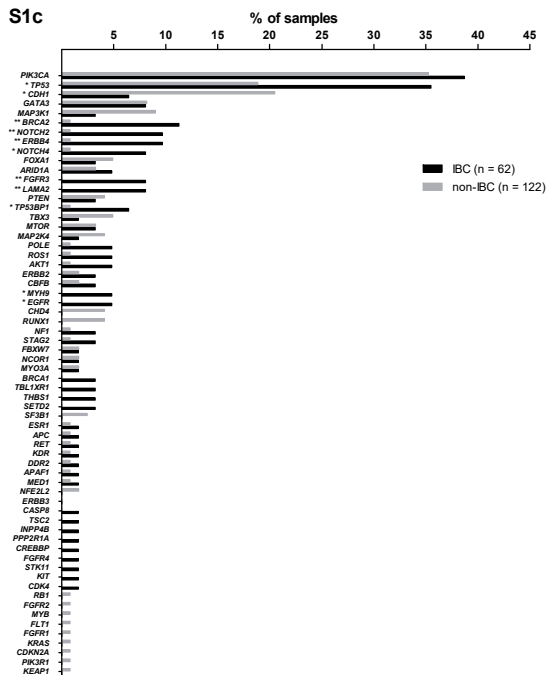

S1d

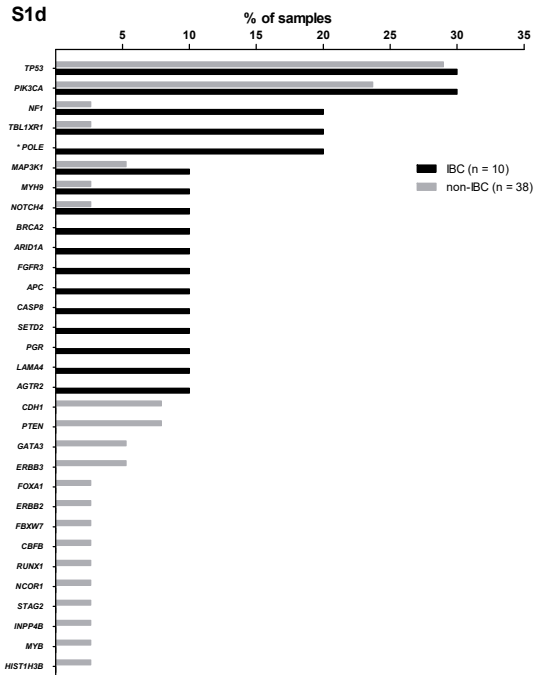

Supplement: Supplementary file 3 — Figure S1. Comparison of somatic mutation frequency between IBC and non-IBC in four subgroups. (a) The percentage of samples with somatic mutation in the TNBC subgroup; (b) the percentage of samples with somatic mutation in the HR–/HER2+ subgroup; (c) the percentage of samples with somatic mutation in the HR+/HER2– subgroup; (d) the percentage of samples with somatic mutation in the HR+/HER2+ subgroup. The gray bars indicate non-IBC, the black bars indicate IBC; *p < 0.05, **p < 0.01, ***p < 0.001. (PDF 52 kb) [file 13058_2018_1007_MOESM3_ESM.pdf]

S2a

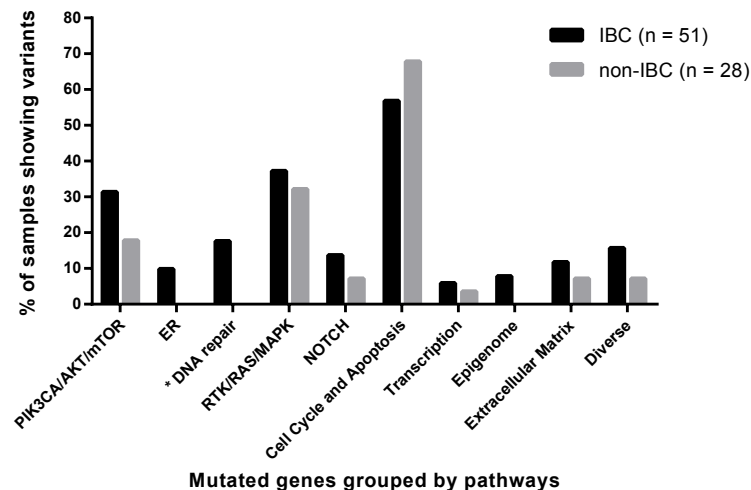

S2b

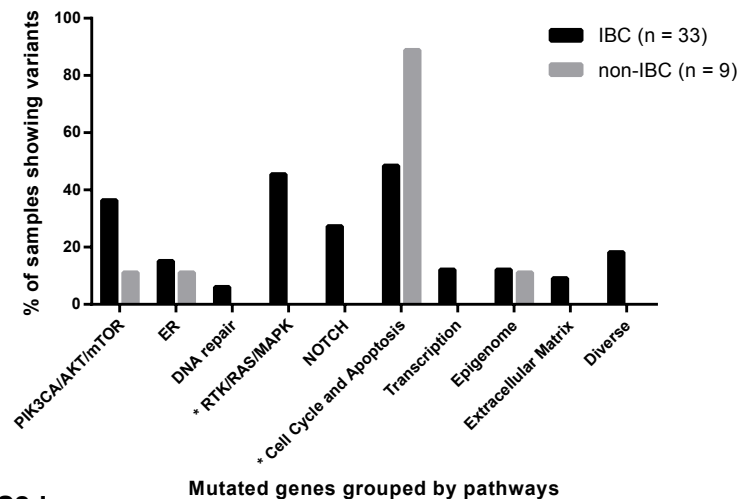

S2c

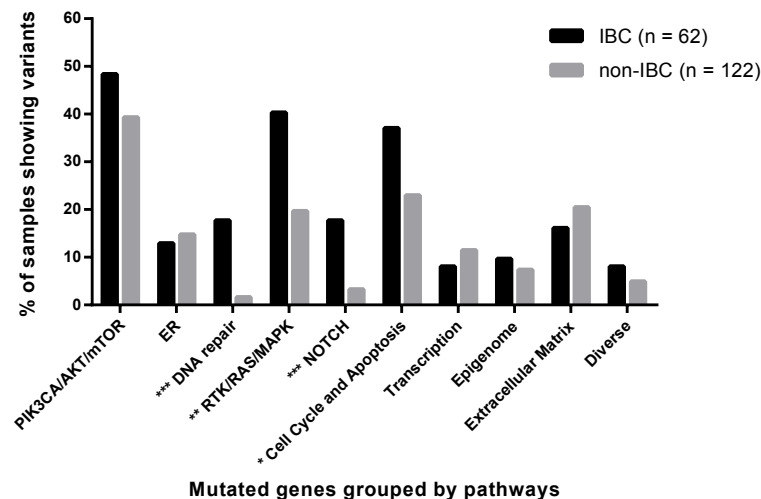

S2d

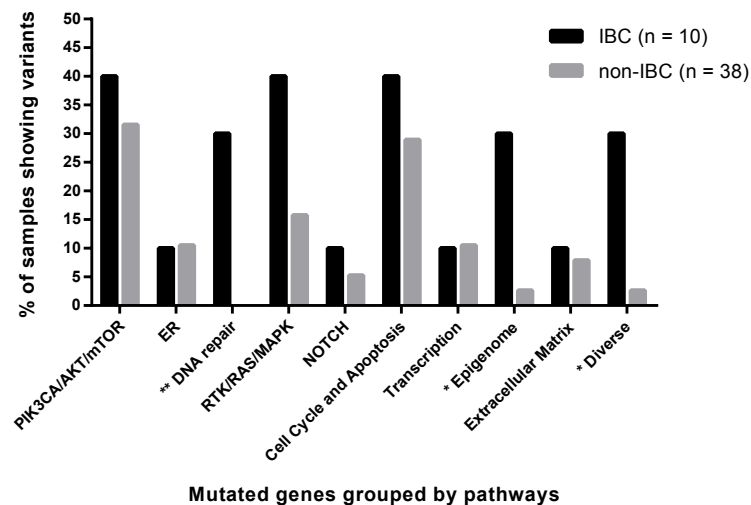

Supplement: Supplementary file 4 — Figure S2. Comparison of biological pathway between IBC and non-IBC in four subgroups. (a) The percentage of samples with alteration on 10 biological pathways in the TNBC subgroup; (b) the percentage of samples with alteration on 10 biological pathways in the HR–/HER2+ subgroup; (c) the percentage of samples with alteration on 10 biological pathways in the HR+/HER2– subgroup; (d) the percentage of samples with alteration on 10 biological pathways in the HR+/HER2+ subgroup. The gray bars indicate non-IBC, the black bars indicate IBC; *p < 0.05, **p < 0.01, ***p < 0.001. (PDF 41 kb) [file 13058_2018_1007_MOESM4_ESM.pdf]

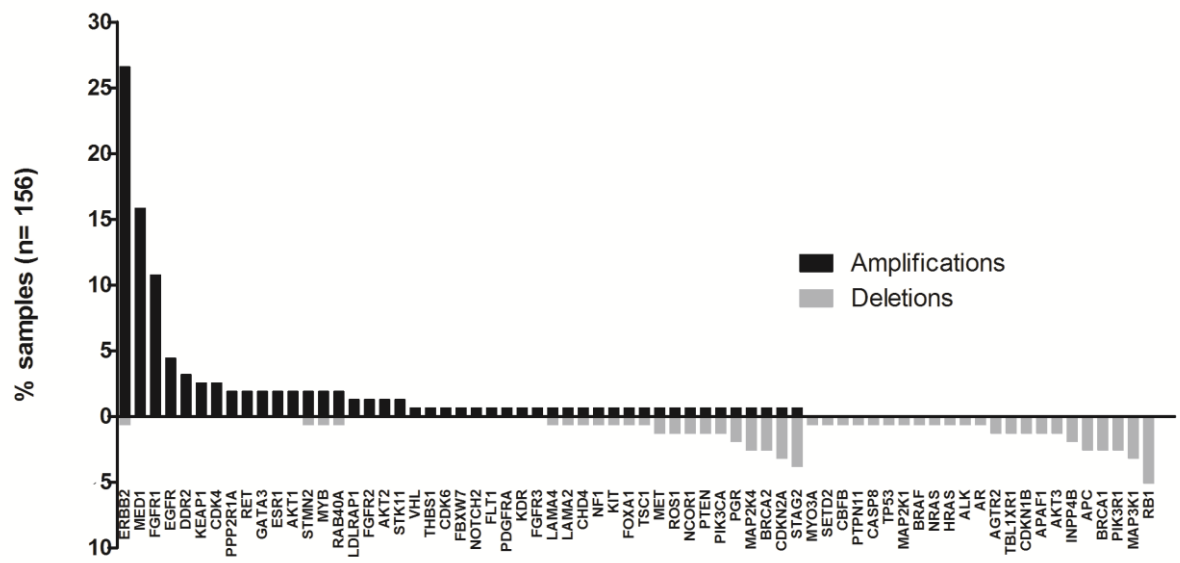

Supplement: Supplementary file 5 — Figure S3. DNA copy number alterations in the IBC cohort. The genes with DNA copy number alterations are grouped along the x axis, the percentage of samples with DNA copy number alterations shown on the y axis, DNA amplifications are indicated by black bars above the x axis, and DNA deletions are indicated by gray bars below the x axis. (PDF 164 kb) [file 13058_2018_1007_MOESM5_ESM.pdf]

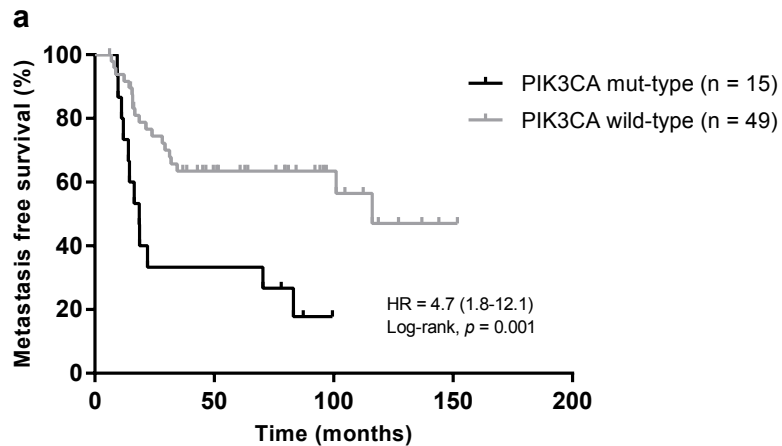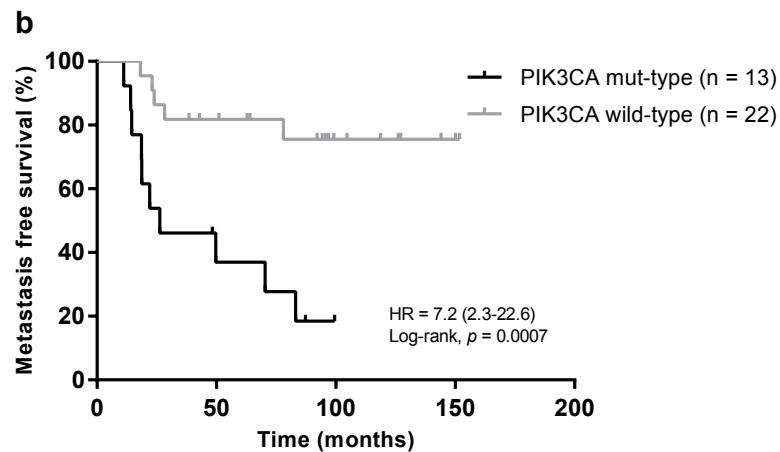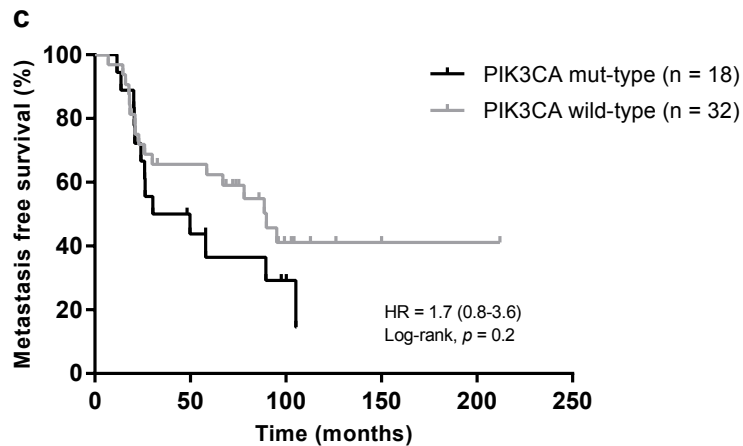

Supplement: Supplementary file 6 — Figure S4. MFS curves stratified by PIK3CA mutation in three subgroups of IBC patients. (a) Kaplan-Meier estimates of MFS according to PIK3CA mutations in patients of the HR– subgroup, (b) Kaplan-Meier estimates of MFS according to PIK3CA mutations in patients of the HER2+ subgroup, (c) Kaplan-Meier estimates of MFS according to PIK3CA mutations in patients of the HR+ subgroup. (PDF 42 kb) [file 13058_2018_1007_MOESM6_ESM.pdf]
